# HIV virological non-suppression and factors associated with non-suppression among adolescents and adults on antiretroviral therapy in northern Ethiopia: a retrospective study

**DOI:** 10.1186/s12879-019-4732-6

**Published:** 2020-01-02

**Authors:** Abraham Aregay Desta, Tewolde Wubayehu Woldearegay, Nesredin Futwi, Gebrecherkos Teame Gebrehiwot, Goyitom Gebremedhn Gebru, Asfawosen Aregay Berhe, Hagos Godefay

**Affiliations:** 1 Tigray Health Research Institute, P. O. Box: 1547, Mekelle, Tigray Ethiopia; 2 Tigray Regional Health Bureau, Mekelle, Tigray Ethiopia

**Keywords:** Viral non suppression, Viral suppression, Viral load, HIV, ART, Ethiopia, Tigray

## Abstract

**Background:**

Despite the benefits of Antiretroviral Therapy (ART), there is a growing concern of treatment failure. This study aimed to assess viral non suppression rate and factors associated with HIV viral non suppression among adolescents and adults on ART in Northern Ethiopia.

**Methods:**

A retrospective cross sectional study was done on 19,525 study subjects. All the data in the database of Tigray Health Research Institute was exported to Microsoft excel 2010 and then data verification and filtration were done before exporting to STATA 14.0 for analysis. Generalized Estimating Equation (GEE) logistic regression was used for statistical modeling of viral non suppression.

**Results:**

A total of 5153 (26.39%; 95%CI (25.77%, 27.02)) patients had no viral suppression despite being on ART. Being male (AOR = 1.27, 95% CI: 1.18, 1.37), 15–19 years of age (AOR = 4.86, 95%CI: 3.86, 6.12), patients from primary hospital (AOR = 1.26, 95%CI: 1.05, 1.52), WHO staging II (AOR = 1.31, 95%CI: 1.10, 1.54), poor ART adherence level (AOR = 2.56, 95%CI: 1.97, 3.33), fair ART adherence level (AOR = 1.61, 95%CI: 1.36, 1.90), baseline CD-4 count of < 200 cells/micro liter (AOR = 1.33, 95%CI: 1.14, 1.54), recent CD-4 count of < 200 cells/micro liter (AOR = 3.78, 95%CI: 3.34, 4.27), regimen types: 1c (AZT-3TC-NVP) (AOR = 1.32, 95%CI: 1.22, 1.44), 2 h (TDF-3TC-ATV/R) (AOR = 1.79, 95%CI: 1.27, 2.52) and declined immunological responses after ART initiation (AOR = 1.45, 95%CI: 1.30, 1.61) were significantly associated with viral non-suppression.

**Conclusions:**

The virological non suppression was high which makes it less likely to achieve the third 90 UNAIDS target. Being male, patients with WHO staging II and poor ART adherence level were significantly associated with viral non suppression. Therefore, intensive adherence support and counseling should be provided. It is also a high time to determine the antiretroviral drugs resistance pattern given the fact that a large number of patients had virological non suppression.

## Background

Globally, among the 36.9 million people living with Human Immunodeficiency Virus (HIV), only 21.7 million were accessing ART in 2017. About 1.8 million people were newly infected with HIV and one million people died from Acquired Immune Deficiency Syndrome (AIDS) related illnesses in 2017 [1]. In Ethiopia, there were 39,140 new HIV infections and 28,650 HIV/AIDS deaths in 2015 [2, 3]. The percentage of people living with HIV among adults (15–49 years) in Ethiopia were 1%. The number of new HIV infections has decreased, from 29,000 to 23,000 in 2018 [4].

According to the UNAIDS report, strong domestic and international investment has stimulated steep declines in HIV infections and deaths from AIDS related illness in eastern and Southern Africa including Ethiopia [5]. However, there are some evidences which showed that the incidence and prevalence of HIV are increasing in Ethiopia and Tigray region in the past three years [6, 7]. According to a study conducted in 2017/18, in urban Ethiopia, among adults aged 15–64 years, the prevalence of HIV ranged from 0.8% (in the Ethiopian Somali region) to 5.7% (in Gambella region) and the same study reported that the prevalence of HIV in Tigray region was 2.7% [6].

Antiretroviral treatment began in 2003 and free ART was launched in Ethiopia in 2005. An estimated 738,976 Ethiopians are currently living with HIV and all of them require ART. However, only 426,000 are currently taking ART [8]. It was estimated that about 79 and 65% of people living with HIV knew their status and were on HIV treatment respectively [4]. The ART coverage in Ethiopia was moderate (52%) [3], 86 and 20% among adult and child population respectively [2]. In a recent report, a total of 64,791 people were living with HIV and there are 39,960 patients on ART care (61.7% coverage) in Tigray [7].

People living with HIV infection require ongoing HIV care and access to medications to maintain continuous maximal virological suppression, allow immune reconstitution, minimize the risk of resistance emergence, prevent HIV-related morbidity and mortality, and prevent transmission of drug resistant HIV mutations [9–12]. Monitoring individuals receiving ART are important to ensure successful treatment, identify adherence problems, and determine whether and which ART regimens should be switched in case of treatment failure. Viral Load (VL) testing should be used aside from the routine testing schedule whenever there is clinical or immunologic suspicion of treatment failure [8].

The World Health Organization (WHO) recommends, where possible the VL of individuals receiving ART be measured every 6 months to detect viral replication and confirm treatment failure [13, 14]. In Low and Middle-Income Countries (LMICs), an elevated or non-suppressed VL Ribo Nuclic Acid (RNA) copies (> 1000 copies/ml in plasma) in a patient who has been on ART for at least six months [14, 15] is an evidence of failing to viral suppression or rebound [16]. This indicates either therapeutic failure and/ or poor adherence to treatment [14, 15]. ART initiation and monitoring in most developing countries was based on the WHO clinical and immunological approach. However, as this approach lacks VL determination, HIV infected adults and children may be at risk of unrecognized virologic failure and viral rebound [17].

Despite the benefits of ART, there is a growing concern about treatment failure, drug resistance, and late drug toxicities associated with long-term use of ART [16, 17], mainly in eastern and southern Africa [18]. Some documents from some African countries have shown that almost 30% of patients develop viral failure within six years after starting ART [19]. A study from Uganda showed young age, poor adherence, and having active TB increased the odds of virological non-suppression while second/third line ART regimens were protective against non-suppression [20]. The emergence of drug resistant viruses limits the treatment option and increases the threat of morbidity and mortality [21].

There was a study in Tigray, however the study was conducted in specific health care facilities with small sample size, which could not show viral non suppression status at region level [22]. In addition to that, factors leading to viral non-suppression may vary across different settings and population groups and hence a context specific data are critical to the implementation of corrective measures against viral non suppression. Thus, this study was aimed to assess viral suppression and factors associated with HIV viral non suppression among adolescents and adults on ART in Tigray, Northern Ethiopia. The finding of this study would help in tracking the progress towards the third of the 90–90-90 UNAIDS plan.

## Methods

### Study setting and data sources

A retrospective cross sectional study was conducted from April, 2015 to March, 2019 at Tigray Health Research Institute (THRI) which is the only center for VL determination specifically for Tigray region and some parts of Northern Ethiopia. Tigray region is the 6th largest by area and the 4th most populous of the 9 Regional States of Ethiopia [23]. ART treatment response follow up by VL determination was started in April 2015 in Tigray region as part of the intensive program of the three 90’s. Blood samples are sent through a sample referral form to the regional laboratory for VL determination from all the health care facilities offering HIV ART services. The samples come from all over the region from both private and government health facilities offering HIV ART services. The sample referral form contains the following information: name of the patient, Medical Registration Number (MRN), Unique ART number, name of the health facility, some demographic data, clinical, treatment, baseline and recent CD-4 count and reason for determining viral load. The source of the data was from all the people living with HIV, enrolled in ART care for at least 6 months whose blood sample was sent for VL determination through standard sample transportation technique to the regional laboratory/THRI from April, 2015 to January, 2019. The study was done among 19,525 patients which had complete data on demographic, clinical, immunological, and viral load in the database of THRI.

### Eligibility criteria

#### Inclusion

- Patients who stayed for at least six months in ART care

#### Exclusion

- Repeated data was excluded and only the recent VL test was used for the single patient
- Subjects younger than 15 years old were excluded from the study

### Sampling procedure

**Fig. 1:**
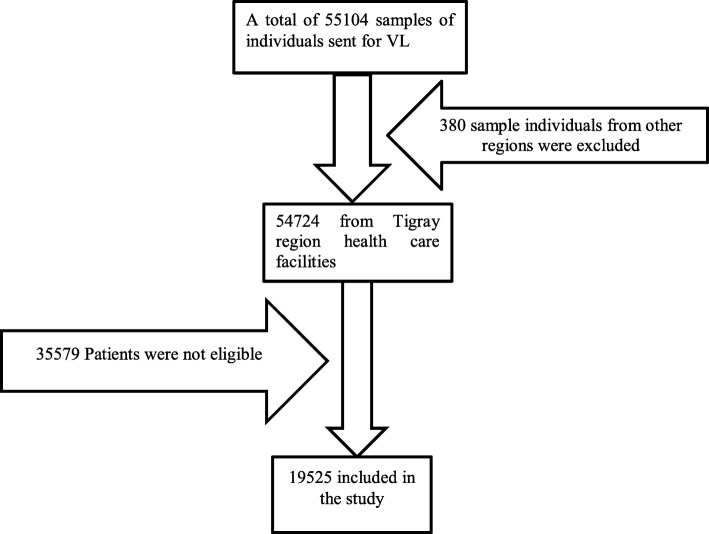
Schematic presentation of sampling procedures

This study was part of the previously published study. To come up with the sample, all records in the database were reviewed and then all samples which came from other regional states were excluded from the study. Finally, all the data that fulfill the eligibility criteria were included in the study (Fig. 1) [24].

### Operational definitions

Good adherence: Drug adherence of 95% or ≤ 2 missed drug doses of 30 doses or ≤ 3 missed drug doses of 60 doses.

Fair Adherence: Drug adherence of 85–94% or 3–5 missed drug doses of 30 doses or 3–9 missed drug doses of 60 doses.

Poor Adherence: Drug adherence of < 85% or ≥ 6 doses of missed ART drug doses of 30 doses or > 9 doses missed ART drug doses of 60 doses.

Viral non-suppression: An elevated VL Ribo Nuclic Acid (RNA) copies of > 1000 copies/ml in plasma in a patient who has been on ART for at least six months.

Declined immunological response: A recent CD4 count of less than the baseline CD4 T-cells count statistically.

Same immunological response: the same recent CD4 count compared to the baseline CD4 T-cells count statistically.

Enhanced immunological response: A recent CD4 count of greater than the baseline CD4 T-cells count statistically.

### Data collection tools and procedure

The data were collected from the database using a data retrieval checklist. The retrieval checklist contains the following variables: name of the health facility (Type, ownership and military or nonmilitary health care facility), some demographic data (age and sex), clinical (WHO staging and ART adherence), treatment (ART regimen), baseline and recent CD-4 count and reason for determining VL. The dependent variable was VL, which was part of the retrieval checklist. All the data in the database were exported to Microsoft excel 2010 and then data verification and filtration were done before exporting to STATA 14.0.

Laboratory testing methods: RNA extraction and Plasma VL determination were made from samples of plasma of each patient sent to THRI. HIV-1 RNA was extracted from 0.2 ml of plasma using Abbott m2000sp automated sample preparation system (Abbott Molecular, USA) in THRI. Extracted RNAs were measured using Abbott m2000rt quantitative Real Time HIV-1 assay (Abbott Molecular, USA) with HIV-1 RNA detection level of 40 to 10 million copies/ml based on the manufacturer’s procedures. Immunological response after ART initiation was assessed by subtracting the baseline CD-4 count from a recent CD-4 count; categorized as deteriorated, the same, and positively responded mathematically.

### Data quality assurance

Data completeness and consistency was checked in the Microsoft excel. The normality of the data set was checked by histogram and normal probability plots. High and low positive controls were checked before performing patient sample for VL determination in THRI. However CD-4 count quality controls were done based on low, medium and high controls to evaluate run validity in each laboratory, where the CD-4 count was done. The results of the baseline and recent CD-4 count of each client was sent via the request form for VL determination to THRI.

### Data analysis

Analysis was done using STATA-14.0 to estimate the proportion of patients with virological non-suppression, and to identify factors associated with virological non-suppression. Univariate analysis was used to describe the socio-demographic and clinical characteristics of the study population.

The proportion of Virological non-suppression was obtained from the total number of patients with no viral suppression divided by the total number of patients tested. The proportion of viorological non-suppression was further evaluated by age group, sex, adherence levels, pregnancy status, breast feeding status, WHO staging, CD-4 baseline, CD-4 recent, immunological response status, regimen, treatment line, reason for VL test, health facility ownership and health facility level. The outcome variable of viral suppression status was categorized based on WHO categorization for LMICs. Missed values were filtered and excluded for all variables; hence there was no issue of missing values in the final data set. The outcome variable was a dichotomous outcome (1 = VL non suppressed and 0 = VL suppressed). The analytic approach used to model this data was the logistic GEE, which takes into account the correlated nature of the outcome variable. The order of responses within a cluster was arbitrary; therefore it was considered exchangeable and independent correlation structures. The specified probability distribution was binomial with logit link function and the working correlation matrix structure was exchangeable. The covariance matrix was robust estimator and the scale parameter was Person chi-square (χ2). The main effect was the term used to build the reported model, and Kernel was specified for the log quasi-likelihood function.

Statistical significance was considered at *p*-value ≤0.05 (two-sided) in all tests. Bivariate analysis was used to determine the strengths of the association between the independent variables and the outcome variable (virological suppression status). Crude odds ratios (COR) were calculated at the bivariate logistic regression. All significant variables at *P*-value ≤0.05 in bivariate analysis were entered into the multivariable analysis. Multivariable logistic regression was used to identify factors independently associated with viral non- suppression. The predictor variables were declared at *p*-value ≤0.05 in the multivariable analysis. As pregnancy, and breastfeeding status applies to females, both variables were excluded from statistical model building of the multivariable analysis. Variables which have a collinearity effect were removed/ omitted in the statistical modeling of the virological non suppression.

## Results

### Patient characteristics

A total of 19,525 individuals were included in the study. The median age (IQR-Inter Quartile Range) of the study participants was 38 (31–45) years. Females accounted for 65.9% of the study participants. About 11,959 (61.25%) of the VL was determined for routine First VL (6 months or more on ART). Only 95 (0.75%) and 202 (1.57%) were pregnant and lactating mothers respectively.

A total of 18,671 (95.63%) people living with HIV were getting ART service at governmental health care facilities. Almost half of the patients (50.34%) were receiving health care service in the general hospitals. The share of private clinics in the provision of ART care was limited where only 43 (0.22%) patients were getting the service at private clinics.

Most of the patients, 18,517 (94.84%) had a good drug adherence. The median (IQR) of the baseline CD-4 count was 201 (112–341). Similarly, the median (IQR) of the recent CD-4 count was 423 (264–611). Almost half the clients, 9390 (48.09%) were on 1e (TDF-3TC-EFV) regimen. A total of 19,284(98.77%) patients were on first line ART regimen (Table 1).

**Table 1:** Associations of variables with viral non-suppression among HIV infected adolescents and adult patients on ART in Northern Ethiopia. (*n* = 19,525)

Variable	Category	Viral suppression status		Total n (%)	COR(95% CI)	AOR (95% CI)
		Suppressed, n (%)	Non suppressed, n (%)			
Gender	Female	9738 (67.76)	3128 (60.70)	12,866 (65.90)	1 (Ref.)	
	Male	4634 (32.24)	2025 (39.30)	6659 (34.10)	1.36 (1.27, 1.45)^***^	1.27 (1.18, 1.37) ^***^
Pregnant mother	No	9659 (99.19)	3112 (99.49)	12,771 (99.26)	1 (Ref.)	1 (Ref.)
	Yes	79 (0.81)	16 (0.51)	95 (0.74)	0.63 (0.37, 1.08)	
Lactating mother	No	9577 (98.35)	3087 (98.69)	12,664 (98.43)	1 (Ref.)	
	Yes	161 (1.65)	41 (1.31)	202 (1.57)	0.79 (0.56, 1.12)	
Age category	15–19	204 (1.42)	216 (4.19)	420 (2.15)	3.67 (2.97, 4.53) ^***^	4.86 (3.86, 6.12) ^***^
	20–24	391 (2.72)	164 (3.18)	555 (2.84)	1.45 (1.19, 1.78) ^***^	1.96 (1.57, 2.45) ^***^
	25–29	1312 (9.13)	520 (10.09)	1832 (9.38)	1.37 (1.20, 1.57) ^***^	1.79 (1.55, 2.08) ^***^
	30–34	2757 (19.18)	959 (18.61)	3716 (19.03)	1.21 (1.08, 1.35) ^***^	1.46 (1.29, 1.65) ^***^
	35–39	3113 (21.6)	1117 (21.68)	4230 (21.66)	1.24 (1.11, 1.39) ^***^	1.43 (1.27, 1.61) ^***^
	40–44	2749 (19.13)	964 (18.71)	3713 (19.02)	1.21 (1.08, 1.36) ^***^	1.22 (1.08, 1.39) ^***^
	45–49	1587 (11.04)	561 (10.89)	2148 (11.00)	1.22 (1.08, 1.39) ^**^	1.22 (1.06, 1.40) ^**^
	50+	2259 (15.72)	652 (12.6)	2911 (14.91)	1 (Ref.)	1 (Ref.)
Age	Median (IQR)	38 (32–45)	37 (30–44)	38 (31–45)		
Facility ownership	Government	13,713 (95.41)	4958 (96.22)	18,671 (95.63)	1.20 (1.02, 1.41) ^*^	1.09 (0.89, 1.33)
	Non-governmental organization	645 (4.49)	195 (3.78)	840 (4.30)	1 (Ref.)	
	Private	14 (0.10)	0	14 (0.07)	___	
Facility type	Clinic	30 (0.21)	13 (0.25)	43 (0.22)	1.35 (0.69, 2.61)	
	Health center	4191 (29.16)	1491 (28.93)	5682 (29.10)	1.11 (0.97,1.26)	
	Primary Hospital	1428 (9.94)	703 (13.64)	2131 (10.91)	1.53 (1.32, 1.77) ^***^	1.26 (1.05, 1.52) ^*^
	General Hospital	7315 (50.90)	2514 (48.79)	9829 (50.34)	1.07 (0.94, 1.21)	
	Referral Hospital	1134 (7.89)	365 (7.08)	1499 (7.68)	1 (Ref.)	
	Other	274 (1.91)	67 (1.30)	341 (1.75)	0.76 (0.57, 1.02)	
Service provided at military health care facility	No	13,837 (96.28)	5013 (97.28)	18,850 (96.54)	1.38 (1.15, 1.67) ^***^	1.47 (1.15, 1.89) ^**^
	Yes	535 (3.72)	140 (2.72)	675 (3.46)	1 (Ref.)	1 (Ref.)
WHO Staging	I	13,255 (92.23)	4574 (88.76)	17,829 (91.31)	1 (Ref.)	1 (Ref.)
	II	481 (3.35)	294 (5.71)	775 (3.97)	1.77 (1.53, 2.06) ^***^	1.31 (1.10, 1.54) ^**^
	III	248 (1.73)	160 (3.10)	408 (2.09)	1.87 (1.53, 2.29) ^***^	1.12 (0.89, 1.40)
	IV	388 (2.70)	125 (2.43)	513 (2.63)	0.93 (0.76, 1.15)	
Adherence	Poor	123 (0.86)	163 (3.16)	286 (1.46)	3.92 (3.09, 4.96) ^***^	2.56 (1.97, 3.33) ^***^
	Fair	413 (2.87)	309 (6.00)	722 (3.70)	2.21 (1.90, 2.57) ^***^	1.61 (1.36, 1.90) ^**^
	Good	13,836 (96.27)	4681 (90.84)	18,517 (94.84)	1 (Ref.)	1 (Ref.)
Virological test reason	Routine First VL	8910 (62)	3049 (51.17)	11,959 (61.25)	1.41 (1.31, 1.52) ^***^	1.35 (1.25, 1.46) ^***^
	Routine annual VL Test	4856 (33.79)	1176 (22.82)	6032 (30.89)	1 (Ref.)	1 (Ref.)
	Suspected ART failure-Clinical	22 (0.15)	28 (0.54)	50 (0.26)	5.26 (3.00, 9.22) ^***^	3.37 (1.86, 6.12) ^**^
	Suspected ART failure-immunological	38 (0.26)	43 (0.83)	81 (0.41)	4.67 (3.01, 7.26) ^***^	2.19 (1.36, 3.51) ^***^
	Suspected ART Failure initial VL	301 (2.09)	790 (15.33)	1091 (5.59)	10.84 (9.35, 12.56) ^***^	7.62 (6.53, 8.90) ^***^
	Not indicated in the form	245 (1.70)	67 (1.30)	312 (1.60)	1.13 (0.86, 1.49)	
CD4 baseline	< 200 cells/micro liter	6950 (48.36)	2737 (53.11)	9687 (49.61)	1.50 (1.34, 1.68) ^***^	1.33 (1.14, 1.54) ^***^
	200–499 cell/ micro liter	5653 (39.33)	1952 (37.88)	7605 (38.95)	1.32 (1.17, 1.48) ^***^	1.32 (1.15, 1.50) ^***^
	+ 500 cells/ micro liter	1769 (12.31)	464 (9.01)	2233 (11.44)	1 (Ref.)	1 (Ref.)
CD4 baseline	Median (IQR)	206 (116–348)	188 (103–320)	201 (112–341)		
CD4 Recent	< 200 cells/micro liter	1542 (10.73)	1501 (29.13)	3043 (15.59)	5.25 (4.78, 5.77) ^***^	3.78 (3.34, 4.27) ^***^
	200–499 cell/ micro liter	6441 (44.82)	2468 (47.89)	8909 (45.63)	2.07 (1.91, 2.23) ^***^	1.89 (1.73, 2.06) ^***^
	+ 500 cells/ micro liter	6389 (44.45)	1184 (22.98)	7573 (38.79)	1 (Ref.)	1 (Ref.)
CD4 Recent	Median (IQR)	463 (307–648)	309 (180–480)	423 (264–611)		
Regimen	1c (AZT-3TC-NVP)	4147 (28.85)	1685 (32.70)	5832 (29.87)	1.30 (1.21, 1.40) ^***^	1.32 (1.22, 1.44) ^***^
	1d (AZT-3TC-EFV)	1362 (9.48)	545 (10.58)	1907 (9.77)	1.28 (1.15, 1.43) ^***^	1.31 (1.16, 1.48) ^***^
	1e (TDF-3TC-EFV)	7155 (49.78)	2235 (43.37)	9390 (48.09)	1 (Ref.)	1 (Ref.)
	1f(TDF-3TC-NVP)	1559 (10.85)	583 (11.31)	2142 (10.97)	1.20 (1.08, 1.33) ^***^	1.30 (1.16, 1.46) ^***^
	1 g (ABC-3TC-EFV)	5 (0.035)	1 (0.019)	6 (0.03)	0.64 (0.07, 5.48)	
	1 h(ABC-3TC-NVP)	4 (0.028)	3 (0.058)	7 (0.04)	2.40 (0.54, 10.74)	
	2a (ABC-ddl-LPV/R), 2c (TDF-ddl-LPV/R), 2d(TDF-ddl-NFV)& 2 g (TDF-3TC-LPV/r)	8 (0.056)	4 (0.078)	12 (0.06)	1.60 (0.48, 5.32)	
	2f (AZT-3TC-ATV/r)	39 (0.27)	23 (0.446)	62 (0.32)	1.89 (1.13, 3.17) ^*^	1.65 (0.95, 2.86)
	2 h (TDF-3TC-ATV/R)	93 (0.65)	74 (1.436)	167 (0.86)	2.55 (1.87, 3.47) ^***^	1.79 (1.27, 2.52) ^***^
Treatment	First line	14,232 (99.03)	5052 (98.04)	19,284 (98.77)	1 (Ref.)	1 (Ref.)
	Second line	140 (0.97)	101 (1.96)	241 (1.23)	2.03 (1.57, 2.63) ^***^	Omitted
Immunological response after ART initiation	Declined	2133 (14.84)	1406 (27.29)	3539 (18.13)	2.16 (2.00, 2.33) ^***^	1.45 (1.30, 1.61) ^***^
	No change	183 (1.27)	67 (1.30)	250 (1.28)	1.20 (0.90, 1.59)	0.86 (0.64, 1.17)
	Enhanced	12,056 (83.89)	3680 (71.41)	15,736 (80.59)	1 (Ref.)	1 (Ref.)

Notes: ^*^significant at *P*-value of ≤0.05; ^**^ significant at *P*-value of ≤0.01; significant at *P*-value of ≤0.001; Omitted, Collinearity effect was allowed to be omitted in the multivariable analysis

Abbreviations: *3TC* lamivudine; *ABC* abacavir; *AOR* Adjusted Odds Ratio; *ATV/r* atazanavir/ Ritonavir; *AZT* azidothymidine; *CD-4* Cluster of Differentiation 4; *COR* Crude Odds Ratio; *ddl* didanosine; *EFV* efavirenz; *LPV/R* Lopinavir/Ritonavir; *n* number; *NFV* nelfinavir; *NVP* nevirapine; *P*-value, Precession value; *Ref* Referrence; *TDF* tenofovirdisoproxilfumarate

### Proportion of viral suppression and non-suppression

**Fig. 2:**
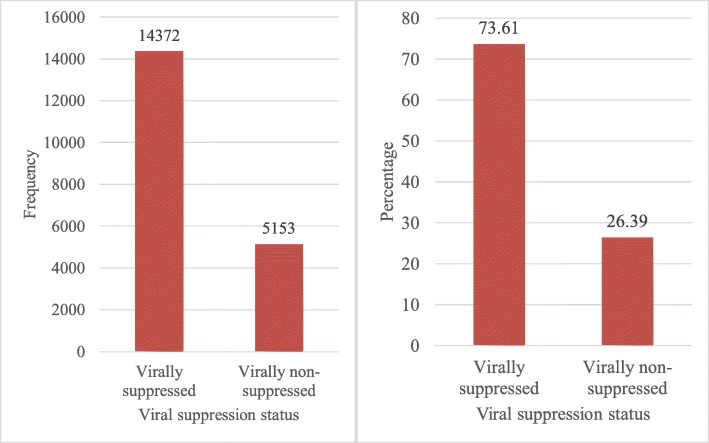
VL suppression status of adolosents and adults patients on ART in Tigray region, North Ethiopia, 2019. (*n* = 19,525)

Out of the total study participants enrolled in ART care, 14,372 (73.61%) had viral suppression. However, a significant number of patients, 5153 (26.39%; 95%CI (25.77%, 27.02)) had no viral suppression (Fig. 2).

### Factors associated with viral non-suppression

The bivariate logistic regression analysis showed that gender, age, patients from governmental health care facilities, patients from primary hospitals, patients from non-military health care facilities, WHO staging II, WHO III, ART adherence, virological test reason, baseline CD4 count, recent CD4 count, 1c (AZT-3TC-NVP) ART regimen, 1d (AZT-3TC-EFV) ART regimen, 1f(TDF-3TC-NVP) ART regimen, 2f (AZT-3TC-ATV/r) ART regimen, 2 h (TDF-3TC-ATV/r) ART regimen, treatment/ ART regimen and immunological response after ART initiation were associated with viral non suppression (Table 1).

After adjusting for possible effects of confounding variables in the multivariable analysis, being male in gender (AOR = 1.27, 95% CI: 1.18, 1.37), age categories from 15 to 19 years (AOR = 4.86, 95%CI: 3.86, 6.12), 20–24 years (AOR = 1.96, 95%CI: 1.57, 2.45), 25–29 years (AOR = 1.79, 95%CI: 1.55, 2.08), 30–34 years (AOR = 1.46, 95%CI: 1.29, 1.65), 35–39 years (AOR = 1.43, 95%CI: 1.27, 1.61), 40–44 years (AOR = 1.22, 95%CI: 1.08, 1.39), 45–49 years old (AOR = 1.22, 95%CI: 1.06, 1.40), patients from primary hospital (AOR = 1.26, 95%CI: 1.05, 1.52) and patients from nonmilitary health care facility (AOR = 1.47, 95%CI: 1.15, 1.89) were significantly associated with viral non-suppression (Table 1)..

Similarly, WHO staging II (AOR = 1.31, 95%CI: 1.10, 1.54), poor ART adherence level (AOR = 2.56, 95%CI: 1.97, 3.33), fair ART adherence level (AOR = 1.61, 95%CI: 1.36, 1.90), test reasons: routine first VL test (AOR = 1.35, 95%CI: 1.25, 1.46), suspected ART failure-clinical (AOR = 3.37, 95%CI: 1.86, 6.12), suspected ART failure-immunological (AOR = 2.19, 95%CI: 1.36, 3.51), suspected ART failure-initial VL > 1000 copies/ml (AOR = 7.62, 95%CI: 6.53, 8.90), baseline CD-4 count of < 200 cells/micro liter (AOR = 1.33, 95%CI: 1.14, 1.54), baseline CD-4 count of 200–499 cell/ micro liter (AOR = 1.32, 95%CI: 1.15, 1.50), recent CD-4 count of < 200 cells/micro liter (AOR = 3.78, 95%CI: 3.34, 4.27), baseline CD-4 count of 200–499 cells/micro liter (AOR = 1.89, 95%CI: 1.73, 2.06), regimen types: 1c (AZT-3TC-NVP) (AOR = 1.32, 95%CI: 1.22, 1.44), 1d (AZT-3TC-EFV) (AOR = 1.31, 95%CI: 1.16, 1.48), 1f (TDF-3TC- NVP) (AOR = 1.30, 95%CI: 1.16, 1.46), 2 h (TDF-3TC-ATV/R) (AOR = 1.79, 95%CI: 1.27, 2.52) and declined immunological responses after ART initiation (AOR = 1.45, 95%CI: 1.30, 1.61) were significantly associated with viral non-suppression (Table 1).

## Discussion

This study was conducted to estimate the proportion of patients with virological non suppression and identify factors associated with viral non-suppression among HIV infected adolescents and adults on ART. The study revealed that 26.39% of them had viral non-suppression and only 73.61% of them have viral suppression. After adjusting for possible effects of confounding variables, gender, age, patients from primary hospital, patients from nonmilitary health care facility, WHO staging II, ART adherence level, viral load test reasons, baseline CD-4 count, recent CD-4 count, regimen types: 1c (AZT-3TC-NVP), 1d (AZT-3TC-EFV), 1f (TDF-3TC- NVP), 2 h (TDF-3TC-ATV/R) and declined immunological responses after ART initiation were significantly associated with viral non-suppression.

The viral suppression rate in this study is low compared to the UNAIDS 90% target for viral suppression on ART [25]. An elevated VL indicates poor adherence or resistance [26–28]. The overall proportion of non-suppression was higher compared to the study conducted in Uganda 11% [20], Systematic review on viral non suppression rate in low or middle-income countries of the pooled estimate had 16% [29], African cohort 9% [30], Nepal 9.92% [31] and Ethiopia 10.4% (self-report) [6] and 19% [1] and 11.5% in Mekelle public hospitals, Tigray, Ethiopia [22]. However, there is a slight agreement with a report from Tigray region, Ethiopia which was 29.8% [6]. These variations of the virological non suppression rate might be due to study design differences which were based on self-report of the patients on ART care [6] or differences in the quality of care in service delivery like counseling and adherence support activities among the different study settings.

This study revealed that the likelihood of developing viral non suppression for male patients was 1.27 times (AOR = 1.27, 95% CI: 1.18, 1.37) more likely when compared with female patients. This finding is in line with studies conducted in Mekelle, Ethiopia [22], worldwide systemic review [32], Nigeria [33], Burkina Faso [34], and Swaziland [35]. The reason why males were prone to viral suppression might be due to low health seeking behavior [33–36]. This study found that the likelihood of developing viral non suppression for patients aged 15 to 19 years was almost 5 times (AOR = 4.86, 95% CI: 3.86, 6.12) more likely when compared with patients aged ≥50 years. A similar study conducted in Uganda [20] and the United States (US) [37] reported that older patients were more likely to achieve viral suppression. Studies have identified alcohol consumption, recreational drug use, low socioeconomic status and age transition contributes to non-adherence and hence virological non suppression among younger age groups [38–41]. Stigma, fear of disclosure, and stress may also affect younger people more than their older counterparts [42]. Young people can be kept in care by using peer supporters, social workers, and training on disclosure among health workers has been reported to contribute to improve ART adherence and hence better virological outcomes [43]. This study revealed that the likelihood of developing viral non suppression for patients from nonmilitary health care facilities was 1.47 times higher (AOR = 1.47, 95% CI: 1.15, 1.89) more likely when compared with patients from military health care facilities. There were no accessed studies on the association of viral non suppression and patients cared at military facilities. But this might be due to better drug adherence among patients receiving ART care in military facilities or as most of the patients who are cared at military facilities might be from the military staff and military staff might be strict in adhering to the ART drugs. The other reason might be due to better nutritional access among the army staff members.

The likelihood of developing viral non suppression for patients in WHO stage II was 1.31 times (AOR = 1.31, 95% CI: 1.10, 1.54) more likely when compared with patients in WHO stage I. However, other studies revealed that WHO stage was not associated with virological failure [22, 44, 45]. This variations might be due to use of different WHO definitions or variation in practice among the different health care providers. Patients who were poorly adherent to the ART drugs were 2.56 times (AOR = 2.56, 95% CI: 1.97, 3.33) more likely to have viral non suppression as compared with patients with good adherence. Other studies have also reported that poor adherence to treatment is positively associated with virological treatment failure [30, 44, 46]. This is because, as the drug concentration decreases in the blood, HIV RNAs might not be suppressed which in turn leads to increase in viral load.

Patients who had a baseline CD4 count of < 200 cells/mm3 were 1.33 times (AOR = 1.33, 95% CI: 1.14, 1.54) more likely to have viral non suppression compared to patients with ≥500 CD4 count at baseline. This study is in line with studies which showed that CD-4 count < 200 cells/mm^3^ was associated with viral load non suppression [44, 45]. The possible reason might be that viral clearance might be slow in patients on ART with low number of CD-4 T-cells count. Patients who had a recent CD4 count of < 200 cells/mm3 were 3.78 times (AOR = 3.78, 95% CI: 3.34, 4.27) more likely to have viral non suppression compared to patients with ≥500 CD4 count. Similar studies revealed that recent low CD-4 count were positively associated with viral non suppression [44, 45, 47, 48]. The possible reason can be, in the presence of immune reconstitution, viral load decreases.

This study showed that the likelihood of developing viral non suppression in patients who were on 1c (AZT-3TC-NVP) regimen were 1.32 times higher (AOR = 1.32, 95% CI: 1.22, 1.44) when compared with patients who were on 1e (TDF-3TC-EFV) regimen. A similar study in Nepal reported that 1e (TDF/3TC/EFV) regimen had a better virological response among Nepalese people living with HIV (PLHIV) [30]. However, other studies reported no association between the ART regimens used and viral non suppression [22, 44]. This variation might be due to genetics and socio demographic variations among the different study settings. All these variables may lay to viral non suppression.

### Strengths and limitations of the study

The study was done in relatively higher sample size with appropriate analysis technique that provides important information regarding sustainability of ART treatment program in Tigray, Northern Ethiopia. Despite these strengths, the study was not without limitation. The VL analysis was based on a single test, hence it may not properly show treatment failure precisely. In addition, due to the nature of a secondary data, the analysis misses some important variables such as the existence of co-infection and grade of ART experience in the HIV infected patients.

## Conclusions

The virological non suppression was high which makes it less likely to achieve the third UNAIDS 90 target. Being male, patients in WHO staging II and poor ART adherence level were significantly associated with viral non suppression. Whereas, patients receiving ART service from military health care facilities, patients with≥500 CD4 baseline count, patients with ≥500 recent CD4 count, 1e regimen and immunological response after ART initiation were significantly associated with viral suppression. Therefore, intensive adherence support and counselling should be provided to achieve the UNAIDS target. It is also high time to determine the antiretroviral drugs resistance pattern given the fact that a large number of patients had virological non suppression.

## Data Availability

The data that support the findings of this study are available from Tigray Regional Health Bureau and Tigray Health Research Institute but restrictions apply to the availability of these data, which were used under license for the current study, and so are not publicly available. Data are however available from the authors upon reasonable request and with permission of Tigray Regional Health Bureau and Institutional Review Board (IRB) of Tigray Health Research Institute. It is possible to contact the IRB through institutional.review.board.thri@gmail.com.

## Abbreviations

3TC: Lamivudine
ABC: Abacavir
AIDS: Acquired Immune Deficiency Syndrome
AOR: Adjusted Odds Ratio
ART: Antiretroviral therapy
ATV/r: Atazanavir/ Ritonavir
AZT: Azidothymidine
CD-4: Cluster of Differentiation 4
CDC: Centers for Disease Control and Prevention
CI: Confidence Interval
COR: Crude Odds Ratio
ddl: Didanosine
EFV: Efavirenz
EPHI: Ethiopian Public Health Institute
GEE: Generalized Estimating Eq.
HIV: Human Immunodeficiency Virus
ICAP: International Centre for Aids Care and Treatment Program
IQR: Inter Quartile Range
LPV/R: Lopinavir/Ritonavir
MRN: Medical Registration Number
N: Number
NFV: Nelfinavir
NVP: Nevirapine
PEPFAR: President’s Emergency Plan For AIDS Relief
PLHIV: People Living With HIV/AIDS
*P*-value: Precession value
Ref: Reference
RNA: Ribonucleic Acid
TDF: TenofovirDisoproxilFumarate
THRI: Tigray Health Research Institute
UNAIDS: United Nations Programme on HIV and AIDS
VL: Viral Load
WHO: World Health Organization

## References

[1] UNAIDS. F A C T S H E E T – W O R L D A I D S D A Y 201 8: 2017 GLOBAL HIV STATISTICS. http://www.unaids.org/sites/default/files/media_asset/UNAIDS_FactSheet_en.pdf. .

[2] Mulu A, Liebert UG, Maier M (2014). Virological efficacy and immunological recovery among Ethiopian HIV-1 infected adults and children. BMC Infect Dis.

[3] Wang H, Wolock TM, Carter A, Nguyen G, Kyu HH, Gakidou E (2016). Estimates of global, regional, and national incidence, prevalence, and mortality of HIV, 1980–2015: the global burden of disease study 2015. Lancet HIV.

[4] UNAIDS. UNAIDS Overview report country Ethiopia 2018. New York: UNAIDS. https://www.unaids.org/en/regionscountries/countries/ethiopia . Accessed on 3 December, 2019.

[5] UNAIDS. UNAIDS Data 2018. New York: UNAIDS. https://www.unaids.org/sites/default/files/media_asset/unaids-data-2018_en.pdf . .

[6] EPHI, PEPFAR, CDC, Westat, ICAP. ETHIOPIA POPULATION-BASED HIV IMPACT ASSESSMENT EPHIA 2017-2018. SUMMARY SHEET: PRELIMINARY FINDINGS. https://phia.icap.columbia.edu/wp-content/uploads/2018/12/3511%E2%80%A2EPHIA-Summary-Sheet_v30.pdf. .

[7] Tigray Regional Health Bureau. HMIS report 2018. Mekelle, Ethiopia: Tigray Regional Health Bureau; 2018.

[8] Federal Democratic Republic of Ethiopia Ministry of health. NATIONAL GUIDELINES FOR COMPREHENSIVE HIV PREVENTION, CARE AND TREATMENT. Version 5, 2017. Addis Ababa: Ethiopian Ministry of Health.

[9] European AIDS Clinical Society. Guidelines Version 8.2, January, 2017. http://www.eacsociety.org/files/guidelines_8.2- english.pdf. Accessed 20 Feburary 2019.

[10] Staszewski S, Miller V, Sabin C, Schlecht C, Gute P, Stamm S (1999). Determinants of sustainable CD4 lymphocyte count increases in response to antiretroviral therapy. AIDS.

[11] Clavel F, Hance AJ (2004). Medical progress: HIV drug resistance. N Engl J Med.

[12] Egger M, Hirschel B, Francioli P, Sudre P, Wirz M, Flepp M (1997). Impact of new antiretroviral combination therapies in HIV infected patients in Switzerland: multicentre study. Br Med J.

[13] WHO. Antiretroviral therapy for HIV infection in adults and adolescents: recommendations for a public health approach, 2010 revision. Geneva: World Health Organization; 2010. 23741771

[14] WHO. Consolidated guidelines on The use of Antiretroviral Drugs for Treating and Preventing HIV Infection: Recommendations for a public health approach. 2013. Geneva: WHO. 24716260

[15] Bennett DE, Bertagnolio S, Sutherland D, Gilks CF (2008). The World Health Organization’s global strategy for prevention and assessment of HIV drug resistance. Antivir Ther.

[16] Guidelines for the use of antiretroviral agents in HIV-1-infected adults and adolescents. Washington: Department of Health and Human Services; 2012. http://aidsinfo.nih.gov/contentfiles/lvguidelines/adultandadolescentgl.pdf . Accessed 11 Feburary 2019.

[17] Hamers RL, Kityo C, Lange JM, de Wit TF, Mugyenyi P (2012). Global threat from drug resistant HIV in sub-Saharan Africa. BMJ.

[18] Bennett DE, Myatt M, Bertagnolio S, Sutherland D, Gilks CF (2008). Recommendations for surveillance of transmitted HIV drug resistance in countries scaling up antiretroviral treatment. Antivir Ther.

[19] Giordano TP, Gifford AL, White AC (2007). Retention in care: a challenge to survival with HIV infection. Clin Infect Dis.

[20] Bulage L, Ssewanyana I, Nankabirwa V, Nsubuga F, Kihembo C, Pande G (2017). Factors associated with Virological non suppression among HIV-positive patients on antiretroviral therapy in Uganda, august 2014–July 2015. BMC Infect Dis.

[21] Ayele G, Tessema B, Amsalu A, Ferede G, Yismaw G (2018). Prevalence and associated factors of treatment failure among HIV/AIDS patients on HAART attending University of Gondar Referral Hospital Northwest Ethiopia. BMC Immunology.

[22] Hailu GG, Hagos DG, Hagos AK, Wasihun AG, Dejene TA (2018). Virological and immunological failure of HAART and associated risk factors among adults and adolescents in the Tigray region of northern Ethiopia. PLoS One.

[23] Wikipedia, the free encyclopedia. https://en.wikipedia.org/wiki/Tigray_Region . Accessed 21 September 2019.

[24] Desta AA, Wubayehu Woldearegay T, Berhe AA, Futwi N, Gebremedhn Gebru G, Godefay H (2019). Immunological recovery, failure and factors associated with CD-4 T-cells progression over time, among adolescents and adults living with HIV on antiretroviral therapy in northern Ethiopia: a retrospective cross sectional study. PLoS One.

[25] UNAIDS. THE SUSTAINABLE DEVELOPMENT GOALS AND THE HIV RESPONSE: Stories of putting people at the centre. http://www.unaids.org/sites/default/files/media_asset/SDGsandHIV_en.pdf . Accessed on 12 March 2019.

[26] Hosseinipour MC, Kumwenda JJ, Weigel R, Brown LB, Mzinganjira D, Mhango B (2010). Second-line treatment in the Malawi antiretroviral programme: high early mortality, but good outcomes in survivors, despite extensive drug resistance at baseline. HIV Med.

[27] Kumarasamy N, Madhavan V, Venkatesh KK, Saravanan S, Kantor R, Balakrishnan P (2009). High frequency of clinically significant mutations after first-line generic highly active antiretroviral therapy failure: implications for second-line options in resource-limited settings. Clin Infect Dis.

[28] Mee P, Fielding KL, Charalambous S, Churchyard GJ, Grant AD (2008). Evaluation of the WHO criteria for antiretroviral treatment failure among adults in South Africa. AIDS..

[29] McMahon JH, Elliott JH, Bertagnolio S, Kubiak R, Jordan MR (2013). Viral suppression after 12 months of antiretroviral therapy in low- and middle-income countries: a systematic review. Bull World Health Organ.

[30] Kiweewa F, Esber A, Musingye E, Reed D, Crowell TA, Cham F (2019). HIV virologic failure and its predictors among HIV-infected adults on antiretroviral therapy in the African cohort study. PLoS One.

[31] Ojha CR, Shakya G, Dumre SP. Virological and Immunological Status of the People Living with HIV/AIDS Undergoing ART Treatment in Nepal. BioMed Research International. 2016; Article ID 6817325, 7 pages. 10.1155/2016/6817325 PMC4980499 27547761

[32] Castilho JL, Melekhin VV, Sterling TR (2014). Sex Differences in HIV Outcomes in the Highly Active Antiretroviral Therapy Era: A Systematic Review. AIDS Res Hum Retroviruses.

[33] Dalhatu I, Onotu D, Odafe S, Abiri O, Debem H, Agolory S (2016). Outcomes of Nigeria's HIV/AIDS Treatment Program for Patients Initiated on Antiretroviral Treatment between 2004–2012. PLoS One.

[34] Penot P, He'ma A, Bado G, Kabore F, Sore I, Sombie D (2014). The vulnerability of men to virologic failure during antiretroviral therapy in a public routine clinic in Burkina Faso. JIAS.

[35] Jobanputra K, Parker LA, Azih C, Okello V, Maphalala G, Kershberger B (2015). Factors associated with Virological failure and suppression after enhanced adherence Counselling, in children, adolescents and adults on antiretroviral therapy for HIV in Swaziland. PLoS One.

[36] Heestermans T, Browne JL, Aitken SC, Vervoort SC, Klipstein-Grobusch K (2016). Determinants of adherence to antiretroviral therapy among HIV-positive adults in sub- Saharan Africa: a systematic review. BMJGlobal Health.

[37] Yehia BR, Rebeiro P, Althoff KN, Agwu AL, Horberg MA, Samji H (2015). The Impact of age on retention in care and viral suppression. J Acquir Immune Defic Syndr.

[38] Reisner SL, Mimiaga MJ, Skeer M, Perkovich B, Johnson CV, Safren SA (2009). A review of HIV antiretroviral adherence and intervention studies among HIVinfected youth. Top HIV Med.

[39] Peltzer K, Pengpid S (2013). Socioeconomic factors in adherence to HIV therapy in low- and middle-income countries. J Health Popul Nutr.

[40] Garvie PA, Wilkins ML, Young JC (2010). Medication adherence in adolescents with behaviorally-acquired HIV: evidence for using a multimethod assessment protocol. J Adolesc Health.

[41] Watson DC, Farley JJ (1999). Efficacy of and adherence to highly active antiretroviral therapy in children infected with human immunodeficiency virus type 1. Pediatr Infect Dis J.

[42] Katz IT, Ryu AE, Onuegbu AG, Psaros C, Weiser SD, Bangsberg DR (2013). Impact of HIV-related stigma on treatment adherence: systematic review and meta-synthesis. J Int AIDS Soc.

[43] van Griensven J, De Naeyer L, Uwera J, Asiimwe A, Gazille C, Reid T (2008). Success with antiretroviral treatment for children in Kigali, Rwanda: experience with health center/nurse-based care. BMC Pediatr.

[44] Bayu B, Tariku A, Bulti AB, Habitu YA, Derso T, Teshome DF. Determinants of virological failure among patients on highly active antiretroviral therapy in University of Gondar Referral Hospital, Northwest Ethiopia: a case–control study. HIV AIDS. 2017;9:153-159. 10.2147/HIV.S139516. eCollection 2017. 10.2147/HIV.S139516 PMC5557910 28848364

[45] Rangarajan S, Colby DJ, Truong GL, Duong BD, Huu HN, Broh TP (2016). Factors associated with HIV viral load suppression on antiretroviral therapy in Vietnam. J Virus Erad.

[46] Casado JL, Sabido R, Perez-Elias MJ (1999). Percentage of adherence correlates with the risk of protease inhibitor (PI) treatment failure in HIV-infected patients. Antivir Ther.

[47] Obiri-Yeboah D, Pappoe F, Baidoo I, Arthur F, Hayfron-Benjamin A, Essien-Baidoo S (2018). Immunologic and virological response to ART among HIV infected individuals at a tertiary hospital in Ghana. BMC Infect Dis.

[48] Mezzaroma I, Carlesimo M, Pinter E, Muratori DS, Di Sora F, Chiarotti F (1999). Clinical and immunologic response without decrease in virus load in patients with AIDS after 24 months of highly active antiretroviral therapy. Clin Infect Dis.

